# Supplementary material for: DrugPTM-Bench: A Large-Scale Dataset for Predictive Modeling of Drug-Induced Cell Type-Specific Protein Post-Translational Modifications
Source: bioRxiv. 2026 Apr 30:2026.04.27.721113. Preprint. [Version 1] doi: 10.64898/2026.04.27.721113 (PMC13142332; doi:10.64898/2026.04.27.721113)
Supplement: Supplement 1 [file NIHPP2026.04.27.721113v1-supplement-1.pdf]

bioRxiv preprint doi: https://doi.org/10.64898/2026.04.27.721113; this version posted April 30, 2026. The copyright holder for this preprint (which was not certified by peer review) is the author/funder, who has granted bioRxiv a license to display the preprint in perpetuity. It is made available under aCC-BY 4.0 International license.

Additional Predictive Tasks Enabled by DrugPTM-Bench

While the primary benchmark task in this work is PTM regulation classification (T1), the multi-dimensional structure of DrugPTM-Bench. The benchmark encompasses pEC50 potency values, full dose-response curves, drug perturbation fingerprints across thousands of sites, and site-level sequence context enabling a family of additional drug discovery relevant tasks. These tasks are formally defined here to establish a community roadmap and invite future contributions. They are not benchmarked in the present work. Each task fills a gap not addressed by existing benchmarks.

T2: pEC50 Potency Regression

In decryptM (51), each PTM site × drug combination with a measurable dose-response is fitted across 11 drug concentrations to yield a pEC50 value. The negative $\log_{10}$ of the molar concentration at which the signal ratio reaches half its maximum effect. The T2 task is to predict this pEC50 from protein sequence context, drug structure, cell line identity, bypassing the need to run the full 11-point dose-response assay. This is fundamentally different from the canonical drug-target affinity benchmarks Davis and KIBA (47), which predict thermodynamic binding affinity (K_d or K_i) measured in cell-free biochemical assays. The pEC50 in DrugPTM-Bench is a *functional cellular potency*: it reflects not only direct binding but also downstream pathway buffering, feedback regulation, and cell-line-specific signaling wiring (51). A drug can have high binding affinity for a kinase yet low pEC50 on a downstream phosphosite if the pathway is buffered by compensatory mechanisms, a distinction that cell-free affinity measurements cannot capture. From a drug discovery perspective, a model that accurately predicts pEC50 at PTM-site resolution could prioritize which drug-site pairs to validate experimentally, reducing the cost of dose-response phosphoproteomics by an order of magnitude. An important limitation is that pEC50 values are only defined for regulated sites (∼47,000 site × drug pairs in DrugPTM-Bench), making the training set smaller than Davis or KIBA, smaller data regimes are very common in computational drug discovery.

T3: Mechanism-of-Action Prediction from PTM Profile

Drugs with the same mechanism of action perturb overlapping sets of PTM sites: kinase inhibitors suppress phosphorylation of their direct substrates and downstream effectors, HDAC inhibitors broadly alter histone and non-histone acetylation, and proteasome inhibitors trigger widespread phosphorylation changes through proteostatic stress (51; 16). A PTM perturbation fingerprint, the vector of signal ratios across all detected sites for a given drug in a given cell line, is therefore mechanistically closer to drug action than transcriptomic signatures, because PTMs are the direct molecular readout of kinase, phosphatase, acetyltransferase, and deacetylase activity rather than a downstream transcriptional consequence. The T3 task is to predict a drug's mechanism class from its PTM fingerprint. Existing MoA prediction benchmarks, including LINCS L1000 (46), MoAble (23), MOASL (24), and the PANACEA DREAM Challenge (13), all use transcriptomic perturbation signatures as input, and none use PTM-level profiles. Mitchell et al. (41) profiled 875 compounds by quantitative proteomics (protein expression, not PTM) and demonstrated MoA clustering, but PTM resolution was not exploited. With 27 drugs spanning eight mechanism classes in DrugPTM-Bench, T3 is a tractable but non-trivial classification problem that could reveal whether PTM fingerprints encode MoA information not captured by transcriptomics.

T4: Drug-Specific PTM Site Sensitivity Ranking

Mass spectrometry-based phosphoproteomics experiments routinely detect thousands of PTM sites per condition, but experimental follow-up (mutagenesis, functional assays, structural studies) can only be performed on a handful of sites (42). A computational model that ranks PTM sites by their predicted sensitivity to a specific drug would directly reduce this experimental bottleneck by prioritizing the most drug-responsive sites for validation. Existing PTM prioritization methods rank sites by intrinsic functional importance: Beltrão et al. (3) used evolutionary conservation and structural context. Ochoa et al. (42) integrated 6,801 proteomics experiments to build a functional landscape of the human phosphoproteome. Lastly, Kennedy et al. (27) developed PTM-centric base editor screens to assess phosphosite functionality at scale experimentally. All of these approaches are drug-agnostic, they rank sites by their general biological importance, not by their sensitivity to a particular perturbation. DrugPTM-Bench enables the first drug-specific PTM site sensitivity ranking benchmark, where the input is a drug fingerprint paired with a protein sequence and all its candidate PTM site contexts, and the output is a ranked list of sites by predicted |signal ratio|. The Johnson et al. kinome substrate

bioRxiv preprint doi: https://doi.org/10.64898/2026.04.27.721113; this version posted April 30, 2026. The copyright holder for this preprint (which was not certified by peer review) is the author/funder, who has granted bioRxiv a license to display the preprint in perpetuity. It is made available under aCC-BY 4.0 International license.

Table 4 Predictive tasks enabled by DrugPTM-Bench. T1 is benchmarked in this work. T2-T4 are defined here as future benchmark tasks. All tasks are formulated at PTM-site resolution.

Task	Setting	Inputs	Target	Why this is new
**T1. PTM Regulation Classification** *(benchmarked in this work)*	3-class classification	PTM-site sequence context, drug identity, dosage, and cell line	Neutral / Up / Down	No existing benchmark jointly models drug identity, dosage, cell context, and direction of PTM regulation at site resolution ((51; 21))
**T2. pEC50 Potency Regression**	Regression	PTM-site sequence context, drug identity, and cell line	Site-level pEC50	Benchmarks such as Davis and KIBA focus on biochemical binding affinity, whereas this task asks for functional cellular potency at PTM-site resolution, which is a different prediction problem ((47; 51))
**T3. Mechanism-of-Action Prediction from PTM Profiles**	Multi-class classification	Drug-induced PTM response profile within a given cell line	Drug mechanism class	Existing benchmarks such as LINCS L1000 and PANACEA are based on transcriptomic response signatures; this task instead uses PTM-level perturbation profiles ((41; 13; 51))
**T4. Drug-Specific PTM Site Sensitivity Ranking**	Learning-to-rank	Drug identity, protein context, and candidate PTM sites on the same protein	Ranked PTM sites by predicted response magnitude	Existing PTM prioritization methods rank sites by intrinsic importance, such as conservation or network relevance, rather than by drug-specific sensitivity ((42; 3; 27; 26))

specificity atlas (26), which maps the sequence preferences of over 300 human Ser/Thr kinases, provides a natural source of sequence-level priors for T4 models targeting phosphorylation sites. Appropriate evaluation metrics for T4 include normalized discounted cumulative gain (NDCG) and Spearman correlation between predicted and observed |signal ratio| rankings per drug-protein pair.

PTM Event Regulation Distribution (T1 data distribution)

bioRxiv preprint doi: https://doi.org/10.64898/2026.04.27.721113; this version posted April 30, 2026. The copyright holder for this preprint (which was not certified by peer review) is the author/funder, who has granted bioRxiv a license to display the preprint in perpetuity. It is made available under aCC-BY 4.0 International license.

Table 5: **Extreme class imbalance in phosphorylation site counts for prediction of PTM regulation at various dosages and time points in the benchmark dataset.**

Cell line	Dosage (μg)	Timepoint (min)	All	Neutral	Up	Down
A431	0	30	174758	174758	non	non
A431	0.03	30	174661	172673	907	1081
A431	0.3	30	174646	172437	837	1372
A431	1	30	174697	172871	1075	751
A431	3	30	174692	171893	1385	1414
A431	10	30	174683	171866	1256	1561
A431	30	30	174709	170714	1456	2539
A431	100	30	174684	169190	1602	3892
A431	300	30	174686	168631	1727	4328
A431	1000	30	174675	168820	1586	4269
A431	10000	30	174686	165355	2246	7085
A549	0	60	274619	273817	397	405
A549	1	60	157947	156730	400	817
A549	3	60	126852	125798	351	703
A549	10	60	157819	156034	584	1201
A549	30	60	126760	125102	519	1139
A549	100	60	157900	155684	747	1469
A549	300	60	126757	124875	577	1305
A549	1000	60	126762	124683	586	1493
A549	3000	60	126677	124502	692	1483
A549	10000	60	126635	124152	799	1684
HeLa	0	30	20367	20367	non	non
HeLa	0	240	45539	42274	1541	1724
HeLa	0	960	12335	12315	12	8
HeLa	0.03	960	6144	6097	23	24
HeLa	0.1	960	6146	6090	21	35
HeLa	0.3	960	6144	6071	33	40
HeLa	1	960	6154	5822	96	236
HeLa	3	960	6148	5907	101	140
HeLa	10	30	20351	17472	1017	1862
HeLa	10	960	6150	5740	167	243
HeLa	30	30	20357	17999	1126	1232
HeLa	30	240	6247	6078	30	139
HeLa	30	960	6142	5724	176	242
HeLa	100	30	20360	19062	806	492
HeLa	100	240	22764	22193	293	278
HeLa	100	960	6141	5644	180	317
HeLa	300	30	20351	18250	944	1157
HeLa	300	240	22726	22015	341	370
HeLa	1000	30	20355	16637	1403	2315
HeLa	1000	240	22760	22188	339	233
HeLa	3000	30	20313	19765	194	354
HeLa	3000	240	22801	22249	314	238
HeLa	10000	30	20355	14766	2218	3371
HeLa	10000	240	22806	21873	461	472
HeLa	30000	30	20347	16846	1278	2223
HeLa	30000	240	22805	21887	510	408
HeLa	100000	30	20339	18876	634	829
HeLa	100000	240	22783	21621	691	471
K562	0	30	83950	83950	non	non
K562	0.01	30	44385	44237	103	45
K562	0.03	30	68663	68358	188	117

Continued on next page

bioRxiv preprint doi: https://doi.org/10.64898/2026.04.27.721113; this version posted April 30, 2026. The copyright holder for this preprint (which was not certified by peer review) is the author/funder, who has granted bioRxiv a license to display the preprint in perpetuity. It is made available under aCC-BY 4.0 International license.

Table 5 – continued from previous page

Cell line	Dosage (µg)	Timepoint (min)	All	Neutral	Up	Down
K562	0.1	30	49108	48702	213	193
K562	0.3	30	73383	72887	322	174
K562	1	30	83653	82543	668	442
K562	3	30	83665	80915	1770	980
K562	10	30	83602	82381	567	654
K562	30	30	83660	82311	552	797
K562	100	30	83625	82191	505	929
K562	300	30	39301	38737	323	241
K562	1000	30	39329	38812	230	287
K562	3000	30	10248	10086	97	65
K562	10000	30	34540	33761	442	337
MDA-MB-175	0	120	5395	5395	non	non
MDA-MB-175	1	120	5392	5336	31	25
MDA-MB-175	3	120	5393	5327	24	42
MDA-MB-175	10	120	5392	5323	13	56
MDA-MB-175	30	120	5392	5326	22	44
MDA-MB-175	100	120	5394	5349	7	38
MDA-MB-175	300	120	5390	5082	51	257
MDA-MB-175	1000	120	5391	5117	36	238
MDA-MB-175	3000	120	5390	4927	91	372
MDA-MB-175	10000	120	5388	4724	193	471
RPMI8226	0	60	24768	24768	non	non
RPMI8226	0	120	23524	23524	non	non
RPMI8226	0	240	20814	20814	non	non
RPMI8226	0	480	17776	17776	non	non
RPMI8226	0	960	26439	26439	non	non
RPMI8226	0.1	60	24758	23782	286	690
RPMI8226	0.1	120	23504	23091	145	268
RPMI8226	0.1	240	20784	20629	88	67
RPMI8226	0.1	480	17764	17577	136	51
RPMI8226	0.1	960	26384	25873	285	226
RPMI8226	1	60	24754	23732	72	950
RPMI8226	1	120	23502	22969	137	396
RPMI8226	1	240	20791	20653	90	48
RPMI8226	1	480	17764	17483	247	34
RPMI8226	1	960	26402	25902	344	156
RPMI8226	10	60	24755	24164	42	549
RPMI8226	10	120	23515	22862	242	411
RPMI8226	10	240	20786	20407	276	103
RPMI8226	10	480	17762	16989	681	92
RPMI8226	10	960	26431	18988	4320	3123
RPMI8226	100	60	24758	23638	63	1057
RPMI8226	100	120	23513	22870	392	251
RPMI8226	100	240	20800	19226	1227	347
RPMI8226	100	480	17764	14219	2101	1444
RPMI8226	100	960	26413	12412	6414	7587
RPMI8226	1000	60	24761	23651	294	816
RPMI8226	1000	120	23515	21892	896	727
RPMI8226	1000	240	20802	18564	1666	572
RPMI8226	1000	480	17766	13761	2321	1684
RPMI8226	1000	960	26416	11652	6594	8170
